# Multi-Matrix Approach for the Analysis of Bicalutamide Residues in Oncology Centers by HPLC–FLD

**DOI:** 10.3390/molecules26185561

**Published:** 2021-09-13

**Authors:** M. Francisca Portilha-Cunha, Teresa I. A. Gouveia, Alicia L. Garcia-Costa, Arminda Alves, Mónica S. F. Santos

**Affiliations:** LEPABE—Laboratory for Process Engineering, Environment, Biotechnology and Energy, Faculty of Engineering, University of Porto, R. Dr. Roberto Frias, 4200-465 Porto, Portugal; mfcunha@fe.up.pt (M.F.P.-C.); up201304237@fe.up.pt (T.I.A.G.); alicia@fe.up.pt (A.L.G.-C.); aalves@fe.up.pt (A.A.)

**Keywords:** antineoplastic drug, bicalutamide, HPLC-FLD, hospital wastewaters, occupational environment, indoor surfaces

## Abstract

Cytostatics are toxic pharmaceuticals, whose presence in surfaces puts healthcare workers at risk. These drugs might also end up in hospital effluents (HWW), potentially damaging aquatic ecosystems. Bicalutamide is a cytostatic extensively consumed worldwide, but few analytical methods exist for its quantification and most of them require advanced techniques, such as liquid chromatography mass spectrometry (LC-MS), which are very complex and expensive for large monitoring studies. Therefore, a simple but reliable multi-matrix high performance liquid chromatographic method, with fluorescence detection, was developed and validated to rapidly screen abnormal concentrations of bicalutamide in HWW and relevant contamination levels of bicalutamide in indoor surfaces (>100 pg/cm^2^), prior to confirmation by LC-MS. The method presents good linearity and relatively low method detection limits (HWW: 0.14 ng/mL; surfaces: 0.28 pg/cm^2^). Global uncertainty was below 20% for concentrations higher than 25 ng/mL (HWW) and 50 pg/cm^2^ (surfaces); global uncertainty was little affected by the matrix. Therefore, a multi-matrix assessment could be achieved with this method, thus contributing to a holistic quantification of bicalutamide along the cytostatic circuit. Bicalutamide was not detected in any of the grab samples from a Portuguese hospital, but an enlarged sampling is required to conclude about its occurrence and exposure risks.

## 1. Introduction

Cytostatics, also known as anticancer drugs or antineoplastic drugs, are pharmaceuticals employed in chemotherapy. Bicalutamide (BICA) is a cytostatic extensively administered worldwide, being used in the treatment of the fourth most common cancer in the world: prostate cancer [1]. However, cytostatics are toxic and not specific for cancerous cells, affecting also healthy tissues, thus posing serious risks on human health. Although BICA has not yet been classified by the International Agency for Research on Cancer (IARC)—which stipulates the potential hazard of cytostatics in relation to their carcinogenicity and reproductive damages—some side effects have been observed, such as severe hepatic damage [2,3].

In addition to treated patients, the exposure of healthcare workers (such as nurses or pharmacy professionals) to cytostatics is particularly relevant, because they have a prolonged and constant exposure to these drugs. Since dermal absorption is the main exposure route [4], surface contamination of indoor settings is the preferred indicator of possible biological uptake (by workers) and of the risk of occupational exposure to cytostatics. Up to now, no exposure limit values have been defined for these drugs. Nonetheless, 100 pg/cm^2^ has been suggested as a threshold for relevant surface contamination—supported on a safe reference value proposed in the literature and on a proposed substance-independent performance-based guideline [5,6,7]. Despite growing concerns and the increase of this research area, there is no analytical method for BICA in indoor surfaces, except the one published by our research team [8]. It is a multiresidue methodology, using liquid chromatography tandem mass spectrometry (LC-MS/MS), which provides an inequivocal confirmation of the presence of these compounds, but it is complex and expensive for large monitoring studies. Moreover, this kind of advanced analytical techniques may be unavailable or unaffordable in healthcare settings/public laboratories, and even more so in low-income countries. Therefore, the development and validation of a simple but reliable methodology, using a less expensive equipment, to allow the screening of a large number of samples prior to confirmation by LC-MS/MS is of utmost importance and it is the main goal of the present study.

On the other hand, there has been an increasing concern regarding the presence of cytostatics in water bodies over the past decade, as 79% of the parent compound is excreted without modifications through urine and feces to hospital and urban wastewaters [9]. Given that hospitals and oncology centers are main focal points of cytostatics’ usage, they are most likely hotspots for the release of these drugs to the environment. BICA is once again one of the most relevant cytostatics in this context, since it was demonstrated by our research team that its concentration remains unaltered in the liquid sewage of a wastewater treatment plant (WWTP) [10]. Up to the authors’ best knowledge, there is only one method in the literature for the analysis of BICA in hospital wastewaters (HWW) [11]. It also requires LC-MS equipment, which is highly sensitive and specific, but it is also expensive and involves specialized personnel for its manipulation, among other disadvantages aforementioned. Regarding the levels of BICA in HWW, that study reports a maximum concentration of 77 ng/L [11]. However, much higher concentrations could be expected, taking the example of other cytostatics, for which a large number of monitoring studies exists (e.g., concentrations of 6.83 mg/L for ifosfamide and 1.28 mg/L for 5-fluorouracil in HWW have been reported [12]). Once again, a screening methodology for the analysis of BICA in HWW by liquid cromatography with a less expensive detector would be very useful.

As far as it is known, liquid chromatography coupled to less selective detectors (e.g., ultraviolet, photodiode array and fluorescence detectors) have only been applied to the analysis of BICA in pharmaceutical formulations and biological fluids. The reported limits of detection are in the range of 5 and 146 ng/mL, which are relatively high [13,14,15,16,17]. Furthermore, the studies lack a full validation procedure, including the estimation of the global uncertainty associated to the results.

In short, the development of an analytical method, able to detect and quantify bicalutamide in different matrices, as wastewaters and indoor surfaces, using a simple and less expensive equipment, could be extremely useful for the on-site detection of cytostatics’ contamination (as a trigger of high concentrations for further confirmation).

Therefore, the aim of this work is to develop and validate a liquid chromatography with fluorescence detector (HPLC–FLD) methodology to assess BICA contamination in oncology facilities. In this sense, two different applications were considered: (i) analysis of BICA in HWW; and (ii) analysis of BICA on relevant surfaces. This will further contribute to a holistic assessment of the exposure risk to BICA in different environmental matrices of the cytostatic circuit.

## 2. Materials and Methods

### 2.1. Chemicals and Reagents

BICA analytical standard of ≥98% purity was acquired from Sigma-Aldrich (St. Louis, MO, USA), as was formic acid. Acetonitrile (ACN), Milli-Q water (ultrapure water—UPW) and methanol (MeOH) were supplied by Merck (Darmstadt, Germany). ACN was of HPLC grade, while Milli-Q water and MeOH were of LC–MS grade. Stock standard solutions were prepared at a concentration of 100 μg/mL in ACN, while working solutions were prepared at 2.5 μg/mL in UPW.

### 2.2. Safety Considerations on Cytostatic Drugs Handling

Exhaustive controls on handling procedures, storage conditions and safety rules were followed for the preparation of standards, as specified by the manufacturers. All procedures involving the handling of BICA were accomplished in a safety hood with vertical laminar airflow, whose working surface was protected by an absorbent paper. All the materials in contact with BICA were cleaned with isopropanol and the dischargeable materials were treated as hazardous waste.

### 2.3. Sample Preparation: Wastewaters

The developed methodology aims to perform a direct analysis of real HWW, without a pre-concentration step. Therefore, samples were only filtered using 0.45 μm nylon membrane filters (Whatmann, Sigma-Aldrich, St. Louis, MO, USA), after adjusting the pH between 5–7, if necessary, with HCl 0.1 M.

### 2.4. Sample Preparation: Surface Extracts

Four model surfaces were assessed for being common surfaces in hospital occupational environments: melamine-coated wood (MCW), phenolic compact (made of phenolic resins and cellulosic fibers) (PC), steel 304 (S304) and steel 316 (S316). Wipe sampling and extraction were based on a previously optimized method for multitarget analysis of cytostatics by LC–MS/MS [8]. Briefly, wipe sampling was performed on 100 cm^2^ of each surface to desorb the cytostatic from the surfaces—each surface was wiped with ¾ of one gauze (commercial gauze: 10 cm × 20 cm, 70% viscose and 30% polyester, 30 g/m^2^) embedded in 2 mL isopropanol (using each ¼ to wipe in a different direction: horizontal, vertical, diagonal) and the remaining ¼ of dry gauze was used to pull the solvent that may have remained on the surface. All gauze parts were placed in a 50 mL Falcon tube and BICA was extracted as follows: 1 mL ACN (extraction solvent) was added; the content was shaken in an ultrasonic bath for 20 min; the organic solvent was recovered from the gauze, transferred to a vial and slowly evaporated to dryness under nitrogen gas; and reconstituted in 200 μL UPW.

### 2.5. Instrumental Analysis

The analyses were carried out in a HPLC (Hitachi; Tokyo, Japan) equipped with an Autosampler L-2200, a Pump L2130 and a fluorescence detector L-2480. Data was acquired and processed using EZChrom Elite software package (Version 3.1.6). Separation was performed with a Pursuit XRs Ultra C18 column (100 mm × 2.0 mm ID, particle size 2.8 µm; Varian). Injection volume was 99 μL.

**Figure A1:**
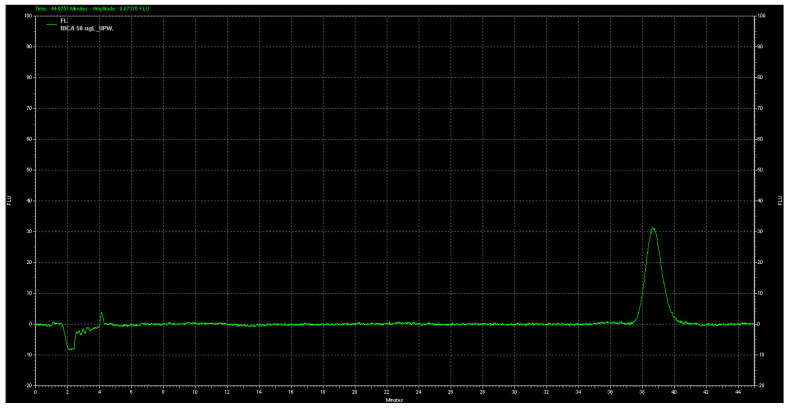
HPLC–FLD chromatogram of a 50 ng/mL BICA standard in ultrapure water, using the method’s final conditions: 0.2 mL/min as flow rate; 65:35 UPW:ACN, both acidified with 0.1% formic acid, as mobile phase; and 272/318 nm as excitation/emission wavelengths.

**Figure A2:**
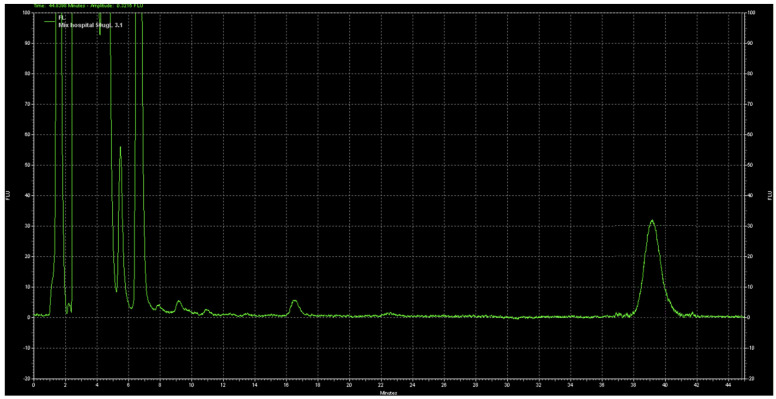
HPLC–FLD chromatogram of a hospital wastewater spiked with 50 ng/mL BICA, using the method’s final conditions: 0.2 mL/min as flow rate; 65:35 UPW:ACN, both acidified with 0.1% formic acid, as mobile phase; and 272/318 nm as excitation/emission wavelengths.

The analytical response of BICA by HPLC–FLD was studied at different working conditions, aiming at selecting the most favorable ones for the target purpose. The following parameters were studied: (i) reconstitution solvent (UPW, MeOH or ACN); (ii) flow rate (0.1, 0.2 or 0.4 mL/min); (iii) mobile phase composition (from 50/50 UPW/ACN to 70/30 UPW/ACN, both organic and aqueous phases acidified with 0.1% formic acid); and (iv) excitation and emission wavelengths (combination of 262, 272 or 282 nm excitation wavelengths with 318, 328 or 338 nm emission wavelengths). Final conditions were: UPW as reconstitution solvent; 0.2 mL/min as flow rate; 65:35 ultrapure water:acetonitrile (*v*/*v*, %), both acidified with 0.1% formic acid, as mobile phase; and 272/318 nm as excitation/emission wavelengths. Statistical analysis was performed to compare the averages of replicate experiments during the optimization of the analytical conditions (*t*-student test). Chromatograms of a 50 ng/mL BICA standard in ultrapure water and in hospital wastewater, obtained under the final conditions, are displayed in Appendix A (Figure A1 and Figure A2, respectively).

#### 2.5.1. Validation Procedure: Quality Control/Quality Assurance

Calibration was performed over a concentration range from 2.5 to 500 ng/mL using 10 calibration points. Statistical analysis was performed to assess the linearity of the calibration curve (Mandel test). All analytical standards were injected in triplicate, and the instrumental detection limit (IDL) and the instrumental quantification limit (IQL) were determined for a Signal-to-Noise ratio of 3 and 10, respectively, and were considered the highest IDL and IQL calculated from each concentration level. Intra-assay precision (repeatability) was assessed by three consecutive injections of the 25, 50 and 100 ng/mL standard solutions and inter-assay precision (intermediate precision) was determined by measuring the same standard solutions in three different days. Intermediate precision of the extracted samples was also assessed, by measuring the analytical response variability for three sample extracts, obtained independently, under the same conditions.

Accuracy for the analysis of BICA in wastewaters by HPLC–FLD was evaluated by spiking a real sample with a concentration of 50 ng/mL of BICA and comparing the analytical response with the one obtained in UPW at the same concentration level. The real wastewater was also directly analyzed by HPLC–FLD to account for the amount of BICA in the original sample (blank sample). A hospital effluent was used as wastewater matrix and experiments were performed in triplicate and recoveries were calculated according to Equation (1):

%R = (Mm − M0)/Ms × 100,(1)

where Mm is the mass of cytostatic measured in the sample from a recovery test, M0 is the mass of cytostatic in the original water (without spiking) and Ms is the spiked mass.

Regarding accuracy for the analysis of BICA in surfaces, 100 cm^2^ of each surface was spiked with 40 µL of a 250 ng/mL solution containing BICA, to achieve a final concentration of 50 ng/mL in the extract. Blank samples were obtained by performing the same sampling and extraction procedure, but without the spiking step. Experiments were performed in triplicate and recoveries were calculated according to Equation (1).

Matrix effects of wastewaters were evaluated by comparing the signal obtained for fortified UPW with the signal for the hospital effluent. For surface samples, matrix effects were analyzed by spiking extracted blank samples, to once again achieve a final concentration of 50 ng/mL.

#### 2.5.2. Global Uncertainty of the Method

The bottom-up approach proposed by the International Organization for Standardization and adopted by EURACHEM-CITAC Guide [18] was applied to estimate the global uncertainty associated with the quantification of BICA in surfaces or wastewaters by HPLC–FLD. Four sources of uncertainty were considered: the uncertainty associated with the preparation of standards (estimated using the error propagation law for the different dilution steps from the stock standard solution); the uncertainty associated with the calibration curve (calculated for the different concentration levels of the standards); the uncertainty associated with the precision of the chromatographic method (estimated as the average result of relative standard deviation for the different concentration levels of intermediate precision assays); and the uncertainty associated with the accuracy (calculated as the average percent recovery obtained within all the experiments). Since the latter source of uncertainty would be different depending on the matrix, different global uncertainties were estimated for surfaces and wastewaters. Detailed equations can be found in Appendix B.

### 2.6. Application

The method developed and validated was applied to 15 wastewater samples from a Portuguese hospital. These samples were collected from five different discharge points within the facility and in three different days.

It was further applied to 28 wipe samples from workplace surfaces from preparation and administration units of cytostatics. Sampling was performed on two weekdays, in the first hours of the working journey. The sampled locations were identified as potentially contaminated for being more frequently handled or touched by healthcare workers, based on direct and passive observations of daily practices.

## 3. Results and Discussion

### 3.1. Optimization of the Chromatographic Method

To select the best solvent for BICA quantification through the present method, the first assays consisted of injecting 1 μg/mL standards using UPW, ACN and MeOH as solvents. The excitation/emission wavelengths were set at 272/328 nm, based on information from the literature [16,17] and the mobile phase was defined as a binary mixture of 50:50 (UPW + 0.1% HCOOH):(CAN + 0.1% HCOOH) at a 0.1 mL/min flow. BICA’s analytical response in UPW was ten-fold higher than in organic solvents (data not shown) and, thus, UPW was chosen to be used as solvent for sample reconstitution.

However, when a real HWW was directly injected, interferences from the real matrix were found in the vicinity of BICA’s retention time. At this point, it was decided to study different working flows (ranging from 0.1 mL/min to 0.4 mL/min) and different percentages of mobile phase (increasing the aqueous phase from 50% to 70%), aiming at avoiding any matrix interference in the quantification of BICA by HPLC–FLD. On one hand, increasing the flow rate of the mobile phase led to shorter runs, where the solvent and matrix effects were curtailed, but BICA’s peak was harder to isolate from the interference peaks. On the other hand, increasing the aqueous phase in the mobile phase led to longer runs, but allowed the stabilization of the baseline after the elution of matrix interferences, which permitted the isolation of BICA’s peak. By increasing the aqueous phase to 65% and simultaneously changing the flow rate to 0.2 mL/min, BICA no longer coeluted with other compounds present in the matrix of HWW. In fact, chromatographic signals from matrix interferences were manifested mainly in the first 10 min of the run, while BICA’s peak was visible at 37 ± 2 min after the implementation of the new/final conditions. Similarly, BICA did not coelute with other compounds present in the matrices of surface extracts (from the four model surfaces assessed), when injected and analyzed using the final conditions. A relative standard deviation of the retention time of 5.4% was found, which is acceptable at such high retention times. Nevertheless, an analytical standard was injected between each four runs to ensure the unequivocal identification of BICA.

**Figure 1:**
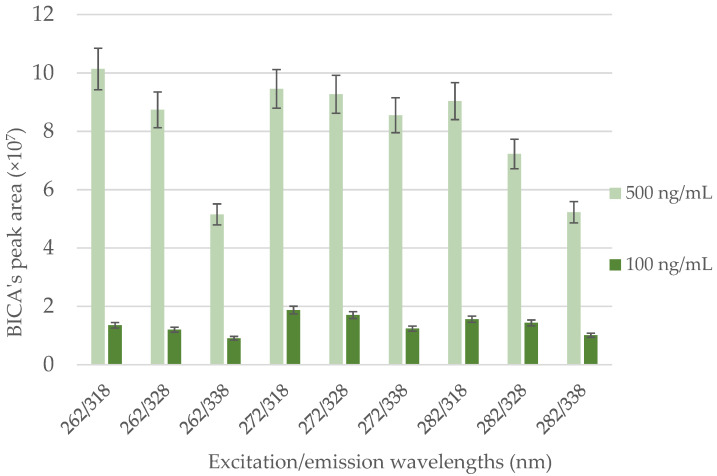
Peak areas obtained for two BICA standards in ultrapure water (100 and 500 ng/mL) by HPLC-FLD at the different combinations of excitation/emission wavelengths (nm).

Then, different excitation/emission wavelengths were tested, aiming at maximizing the chromatographic response of BICA. The only two methods found in the literature for the analysis of BICA by HPLC-FLD report the use of 272 and 328 nm for excitation and emission wavelengths, respectively [16,17]. Therefore, the fluorescence response of BICA was analyzed in the vicinity of this excitation/emission wavelength pair. Two standards of different concentrations (100 ng/mL and 500 ng/mL) were injected (triplicate) in a combination of three excitation (262, 272, 282 nm) and three emission (318, 328, 338 nm) wavelengths, resulting in a combination of nine tests for each standard. As displayed in Figure 1, the combination 262/318 nm was considered statistically similar to the combination 272/318 nm for the highest standard (500 ng/mL). However, 272/318 nm was the combination with statistically higher areas for the lowest standard (100 ng/mL), using the *t*-student test (significance level of α = 0.01). Therefore, 272/318 nm was defined as the optimum combination of excitation and emission wavelengths for the quantification of BICA by HPLC–FLD. Moreover, this set of excitation and emission wavelengths fits the target purpose of analyzing BICA at relevant concentration ranges in HWW and surfaces, as detailed in Section 3.2.1. Hence, the final conditions were: UPW as reconstitution solvent; 0.2 mL/min as flow rate; 65:35 ultrapure water:acetonitrile (*v*/*v*, %), both acidified with 0.1% formic acid, as mobile phase; and 272/318 nm as excitation/emission wavelengths.

### 3.2. Validation Parameters of the Chromatographic Method

#### 3.2.1. Linearity and Limits of Detection and Quantification

Good linearity was achieved for BICA in UPW using the optimized parameters for HPLC–FLD analysis, in a range of 2.5–500 ng/mL. The regression equation is given by: Area = (1.900 ± 0.007) × 10^5^ × Concentration (ng/mL) + (−2.247 ± 1.239) × 10^5^, with a very good R^2^ (0.9999). The Mandel test was applied to evaluate the linearity by comparing the test value (*F*test) with the corresponding value of the *F*-distribution with 1 and *N*-3 (*N* is the number of calibration points = 10) degrees of freedom at the significance level of α = 0.01. It was found that the calibration function is linear, i.e., no significantly better fit was obtained by the second-degree calibration function. The IDL and IQL, determined for a Signal-to-Noise ratio of 3 and 10, respectively, were 0.14 and 0.45 ng/mL. The method detection limit (MDL) is the same as the IDL (0.14 ng/mL) for wastewater analysis, whereas the MDL for surface analysis is 0.28 pg/cm^2^, taking into account the concentration factor of the extraction step and assuming 100% recovery. The detection and quantification limits of this method are ten to a thousand times lower than those reported in the few methodologies found in the literature for the analysis of BICA by liquid chromatography coupled to less expensive detectors [13,14,15,16,17].

Therefore, concerning water matrices, this method can be a useful tool to directly quantify BICA without an extraction step. As previously referred, some cytostatics have been found in HWW in concentrations up to 6.83 mg/L [12], much higher than the IQL obtained in this study. Still, information regarding BICAs monitorization in wastewaters is scarce and a simple method such as HPLC–FLD can be an easier and less expensive alternative than LC–MS/MS, which is the most common methodology currently employed. However, whenever BICA is identified by HPLC–FLD, a confirmation by LC–MS should occur.

Regarding surface contamination, the “as low as reasonably achievable” (ALARA) principle is the best standard to reduce occupational exposure to cytostatics, since no exposure limit values have been defined for these drugs. Hence, it is important to develop methods with low sensibilities. Nonetheless, 100 pg/cm^2^ has been suggested as a threshold for relevant surface contamination and it should be quantifiable by developed methods. In the present method, that value corresponds to the 50 ng/mL standard, taking into account the sampled area of 100 cm^2^, while the calibration curve goes up to 1000 pg/cm^2^. Although some LC–MS/MS methods have achieved MDLs as low as 0.01 pg/cm^2^ for other cytostatics [19], the only method found for analysis of surface contamination with BICA displays a MDL of 0.1 pg/cm^2^ [8]. This is lower than the MDL of the present methodology (0.28 pg/cm^2^), but it was obtained by LC–MS/MS [8]. Hence, the present HPLC–FLD method fits the requirements for properly assessing surface contamination with BICA, especially in low-income countries or in healthcare settings/public laboratories, where advanced instrumental techniques may be unavailable or unaffordable.

#### 3.2.2. Precision and Accuracy

Intra-day precision was evaluated by three consecutive injections of the 25, 50 and 100 ng/mL standard solutions which resulted in coefficient of variations of 5.6%, 4.0% and 1.5%, respectively. Although the latter is slightly above 10%, the obtained values show the FLD response is precise. Furthermore, good inter-day precision was observed through the injection of the same standard solutions (25, 50 and 100 ng/mL) on three different days. The results were 6.9%, 4.6% and 2.3%, respectively.

Regarding the HWW, 98 ± 17% BICA was recovered from three independent assays with a spiking step at a concentration level of 50 ng/mL, and with an intermediate precision of 20% (coeficient of variation). Blank samples were prepared and analysed to check whether BICA was present in the original samples (without spiking), which would then be taken into consideration for calculating the recoveries. However, BICA was not detected in the HWW. The effect of the HWW matrix in the quantification of BICA was almost negligible since this matrix does not emit fluorescence in the retention time of BICA in the conditions defined. The recovery and the precision achieved are satisfactory and acceptable for the intended purpose. Hence, the FLD analysis allows a sufficiently accurate and precise quantification of BICA in hospital effluents.

Concerning the surfaces, 88 ± 5%, 83 ± 2%, 81 ± 3% and 85 ± 2% of BICA was recovered from S304, S316, MCW and PC, respectively. This indicates that BICA’s recovery is surface-independent (mean recovery: 84 ± 4%), under the present extraction conditions and instrumental analysis. In addition, very low matrix effects were observed: −2%, −7%, −9% and 4%, respectively; which shows these matrices do not interfere with the quantification of BICA. Blank samples (without spiking) were prepared and analyzed, and BICA was not detected in any of the four model surfaces. Furthermore, the intermediate precision, obtained from measuring triplicate samples independently extracted under the same conditions, was 5%. These recoveries are very good and suitable for the intended purpose, as is the precision. Hence, the FLD analysis allows an accurate and precise quantification of BICA in surfaces.

### 3.3. Global Uncertainty Associated with Results

**Figure 2:**
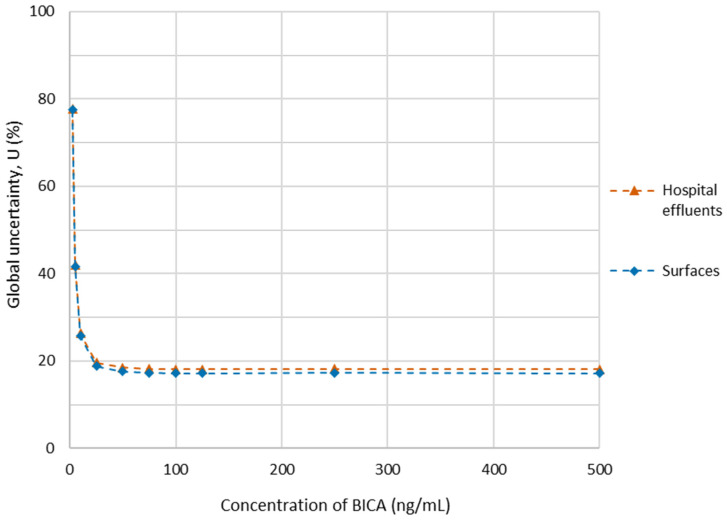
Global uncertainty of the analytical methodology for bicalutamide quantification in hospital effluents and surfaces by HPLC–FLD. Dashed lines are merely illustrative of the data trend. 50 ng/mL corresponds to 100 pg/cm^2^ in the case of surface samples.

In addition to percent recoveries, global uncertainty should also be considered when interpreting results. In fact, this is an important consideration in method validation, mainly when comparing results from different methods. The global uncertainty associated with the present method was estimated, having in consideration the relative weight of the standards preparation, the calibration curve, the precision and the accuracy assays. Figure 2 shows that the global uncertainty is much higher for lower concentrations, as expected. This is verified for both the hospital effluents’ matrix and the surfaces, achieving a maximum of approximately 78% uncertainty for the 2.5 ng/mL standard in both cases (which corresponds to 5 pg/cm^2^ in the case of surface samples). However, for concentrations higher than 25 ng/mL (which corresponds to 50 pg/cm^2^ in the case of surface samples), the global uncertainty is always below 20%, which is an acceptable value.

**Figure 3:**
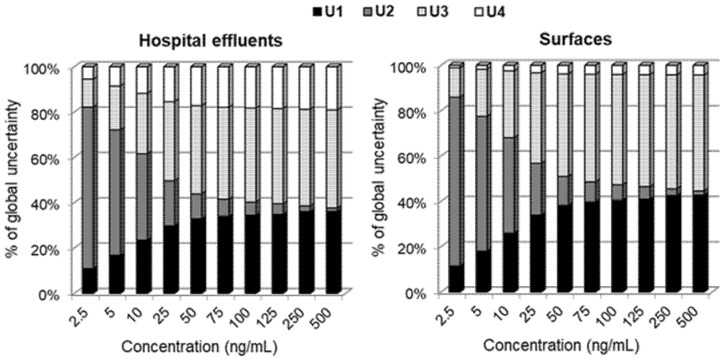
Relative weight of each individual source of uncertainty (bottom-up approach/EURACHEM) for bicalutamide quantification in hospital effluents and surfaces by HPLC–FLD. U1—uncertainty associated with standard preparation; U2—uncertainty associated with the calibration curve; U3—uncertainty associated with precision; U4—uncertainty associated with accuracy. 50 ng/mL corresponds to 100 pg/cm^2^ in the case of surface samples.

Individual contributions of the four sources considered for the global uncertainty are depicted in Figure 3. It shows that, in both the hospital effluents’ matrix and the surfaces, the uncertainty associated with the calibration curve is the major contributor for the error at lower concentrations—thus being the reason for the high global uncertainty values observed in Figure 2. Nevertheless, its contribution to the global uncertainty at higher concentrations is almost negligible, where the uncertainty associated with the preparation of standards and with the precision is much more significant. The uncertainty associated with accuracy is generally low across the entire range of concentrations studied for both matrices. As such, the effect of the type of matrix on global uncertainty is very little (the curves in Figure 2 are almost overlapped). This means that the same uncertainty could be applied for the quantification of BICA in both matrices (surfaces and wastewaters) by the present HPLC–FLD methodology. This further grounds the multitarget character of the present analytical methodology and the possibility of being applied to other occupational environment matrices, which would be an added-value to obtain an integrated knowledge about exposure of Humans to BICA at indoor settings.

### 3.4. Application

The validated analytical methodology was applied for the analysis of HWW samples as well as workplace surfaces from preparation and administration wards of a hospital. BICA was not detected in any of the samples; however, it is important to highlight that only a few samples were analyzed and only grab samples were considered in the sampling scheme (24-h composite samples may provide more information than discrete samples in the particular case of wastewaters). Since a thorough discussion needs to be supported by an enlarged monitoring scheme, considering both grab and composite samples, no conclusions can be drawn. As such, a larger sampling scheme is being planned to carry out in the near future.

## 4. Conclusions

A HPLC–FLD method was developed and validated for BICA quantification in different environmental matrices related to oncology centers, namely HWW and indoor surfaces. Using a C18 column (100 mm × 2.0 mm ID, particle size 2.8 µm; Varian) and the final optimized parameters, such as 65:35 (UPW + 0.1% HCOOH):(CAN + 0.1% HCOOH) of mobile phase at 0.2 mL/min and excitation/emission wavelengths set to 272/318 nm, BICA’s retention time was 37 ± 2 min. Good linearity (statistically evaluated through Mandel test), correlation coefficient (R^2^ = 0.9999), method detection limits (hospital wastewaters: 0.14 ng/mL; surfaces: 0.28 pg/cm^2^) and intra- and inter-day precision (RSD < 10%) were achieved. Spiking of HWW and of four model surfaces provided recoveries of 98 ± 17% and 84 ± 4%, and intermediate precisions of 20% and 5% (coefficients of variation), respectively. Hence, this method is suitable to directly quantify BICA in HWW without an extraction step and to assess surface contamination by BICA at relevant concentrations (the threshold value for relevant surface contamination is 100 pg/cm^2^). Furthermore, global uncertainty associated to the results was below 20% for concentrations higher than 25 ng/mL (HWW) and 50 pg/cm^2^ (surfaces), being the contribution of the matrix to global uncertainty very little. Therefore, a multi-matrix assessment could be achieved with this method, thus contributing to a holistic quantification of BICA along the cytostatic circuit.

Moreover, the method was employed to quantify BICA in some HWW samples and workplace surfaces’ samples. The target cytostatic was not detected in any of the grab samples collected; an enlarged sampling campaign is required to conclude about the occurrence of BICA in hospital wastewaters and workplace surfaces, and this is planned for the near future.

## Data Availability

Data sharing not applicable.

## Appendix B. Estimation of the Global Uncertainty Associated to the Analytical Results

The bottom-up approach proposed by the International Organization for Standardization and adopted by EURACHEM-CITAC Guide [18] was applied to estimate the global uncertainty (*U*) associated with the quantification of BICA in surfaces or water matrices by HPLC–FLD. Global uncertainty is thought to be mainly affected by four sources of uncertainty and is expressed as:

(A1) $$U=\sqrt{U1^{2}+U2^{2}+U3^{2}+U4^{2}},$$

where:

1. *U*_1_ is the uncertainty associated with the preparation of standards (estimated using the error propagation law for the different dilution steps from the stock standard solution):
  (A2) $$U_{1}=\sqrt{\sum_{i}\left(\frac{\Delta mi}{mi}\right)}.$$
  Δ*mi*—error associated with the measurement equipment; *mi*—measured value.
2. *U*2 is the uncertainty associated with the calibration curve (calculated for the different mass levels of the standards):
  (A3) $$U_{2}=\frac{sx_{0}}{x_{0}}=\frac{1}{x_{0}}\frac{s_{y/x}}{a}\sqrt{\frac{1}{m}+\frac{1}{n}+\frac{{\left(y_{0}-\bar{y}\right)}^{2}}{a^{2}\sum_{i}{\left(x_{i}-\bar{x}\right)}^{2}}}=\frac{1}{x_{0}}\frac{1}{a}\sqrt{\frac{\sum_{i}{\left(y_{i}-y_{0}\right)}^{2}}{n-2}}\sqrt{\frac{1}{m}+\frac{1}{n}+\frac{{\left(y_{0}-\bar{y}\right)}^{2}}{a^{2}\sum_{i}{\left(x_{i}-\bar{x}\right)}^{2}}}.$$
  *sx*_0_—standard deviation of the concentration; *x*_0_—mass; *a*—slope of the calibration curve (*y* = *ax* + *b*); *m*—number of replicates performed; *n*—number of standards to build the calibration curve; *y*_0_—*y* values calculated by the calibration curve from *x* values; *ȳ*—average of *y_i_* (experimental) values of the calibration curve standards; *x_i_*—concentration of each standard used in the calibration curve; $\bar{x}$—average of *x_i_* values; *y_i_*—experimental values of the calibration curve standards.
3. *U*_3_ is the uncertainty associated with the precision of the chromatographic method (estimated as the average result of relative standard deviation for the different concentration levels of intermediate precision assays):
  (A4) $$U_{3}=\frac{s}{\bar{y}\sqrt{n}}.$$
  *s*—standard deviation of experimental *y* values in precision assays; *ȳ*—average of experimental *y* values in precision assays; *n*—number of precision assays.
4. *U*4 is the uncertainty associated with the accuracy (calculated as the average percent recovery obtained within all the experiments):
  (A5) $$U_{4}=\frac{s\left(\eta \right)}{\sqrt{n}}.$$
  *s*(*η*)—relative standard deviation of the average percent recovery; *n*—number of accuracy assays.
